# Dietary Patterns as Characterized by Food Processing Levels and Their Association with the Health Outcomes of Rural Women in East Africa

**DOI:** 10.3390/nu13082866

**Published:** 2021-08-20

**Authors:** Jacob Sarfo, Elke Pawelzik, Gudrun B. Keding

**Affiliations:** Division Quality of Plant Products, Department of Crop Sciences, University of Goettingen, 37075 Göttingen, Germany; epawelz@gwdg.de (E.P.); gkeding@gwdg.de (G.B.K.)

**Keywords:** Africa, dietary patterns, overweight and obesity, rural areas, processed foods, women

## Abstract

Overweight and obesity are rapidly rising in Sub-Saharan Africa including in rural areas. However, most studies focus on urban centers, and have attributed this epidemic to the consumption of processed foods without their clear characterization. This study investigated food intake patterns defined by food processing levels and their association with overweight/obesity in rural areas. Four 24-h dietary recalls, anthropometric measurements, and socio-demographic characteristics were collected from 1152 women in Kenya, Tanzania, and Uganda. The PCA method was used to extract patterns characterized by food processing levels. The association between patterns and overweight/obesity was ascertained with regression models. The overweight/obesity rate was 47%, 42%, 26%, and 38% in Kenya, Tanzania, Uganda, and East Africa (as pooled data), respectively. Several patterns were identified, yet a “plant-based pattern” largely characterized by unprocessed and minimally processed foods and a “purchase pattern” mainly distinguished by highly processed foods were dominant. The “plant-based pattern” was inversely or not associated with overweight/obesity, while the “purchase pattern” had a positive association or no association. A clear distinction on processed foods as healthy and unhealthy should be made based on their nutrient provision to avoid their mischaracterization as unhealthy. Policies to reverse consumption of unhealthy processed foods while promoting healthy ones should be pursued.

## 1. Introduction

Malnutrition, in all its forms, presents a key global challenge to good health outcomes among individuals and populations [1]. Of crucial concern among global nutrition challenges is rising overweight and obesity levels. In 2016, the number of obese adults was estimated to be around 678 million [2]. Shekar and Popkin [3] intimated that more than 70% of the world’s overweight and obese adults reside in low- and middle-income countries, and a global yearly death rate of 4 million is attributed to overweight and obesity [4]. Overweight and obesity—which are a resultant effect of too little energy expenditure to compensate for excessive energy intake—are largely driven by a shift in food intake from nutrient-dense foods to energy-dense foods that are highly concentrated in sugars, fats, salt, and multiple other ingredients. The shift in food intake is what is largely known as the “nutrition transition” [3,5]. Overweight and obesity do not only spur the risks of non-communicable diseases (NCDs) such as type 2 diabetes, cardiovascular diseases, and certain cancers [6,7], but they also unleash dire economic consequences for countries through lost productivity and increased health costs [3].

As a result, over the years, many studies have gauged the possible linkages between a shift in food intake and several health outcomes including overweight and obesity levels. Initial analyses of these linkages relied on using single food items or nutrients, yet the complexity of the human diet requires understanding the interactive effects of both food and nutrients, and not either in isolation [8,9]. Hence, a dietary pattern—referred to in this study as pattern—analysis provides a more useful tool to combine both food and nutrients to describe an entire diet and associate it with health outcomes. Several statistical methods have been used to characterize the patterns of populations, as patterns are directly unmeasurable [8]. Some statistical methods used so far include principal component analysis (PCA), cluster analysis, index analysis, and the reduced rank regression. The PCA and cluster analyses use an *a posteriori* approach to explore data to classify foods/food groups that are correlated to better explain diets or cluster people together based on dietary intake, respectively. The index analysis is based on an *a priori* method where individuals are scored based on predefined diet indexes; such indexes are normally constructed based on knowledge from previous studies [10,11]. The reduced rank regression approach uses both *a posteriori* and *a priori* methods to assess the linear function of foods/food groups that provide significant variation in a set of predetermined response outcomes, usually diet-related diseases [12,13]. Thus far, PCA has been a well-established statistical tool for approximating patterns to describe the diet behavior of a population. In Sub-Saharan Africa (SSA), for example, studies on patterns and their linkage to different health outcomes have been mostly PCA-driven studies [9,14,15,16,17,18].

However, these studies and others have been centered around urban areas to the neglect of rural communities. To the best of our knowledge, only two studies have been performed in SSA focusing on rural women in Tanzania and rural adolescents in South Africa, conducted by Keding et al. [15] and Pisa et al. [16], respectively. Meanwhile, overweight and obesity levels in rural areas are rapidly growing as part of the overall rising levels of this health epidemic in low- and middle-income countries [3,4]. Furthermore, most of these studies that use data reduction methods such as PCA to determine patterns in SSA broadly attribute processed foods to rising overweight and obesity and other NCDs. This notwithstanding, food processing largely provides an avenue for a safe, diverse, adequate, and easily accessible supply of food [19]. Thus far in the literature, processed foods have been classified by their degree of processing, and notable among them is the NOVA classification. The NOVA classification mainly classifies foods based on the extent and purpose of their industrial processing [20]. Indeed, several studies—mostly in high- and middle-income settings—have adopted the NOVA classification to distinguish processed foods to establish their relationship with varied health outcomes [21]. However, in SSA, studies on the characterization of processed foods in pattern assessment are limited, with none for rural settings to the best of our knowledge.

Through this study, we contribute to addressing these literature gaps by employing longitudinal food intake data and the usage of a recent food processing classification by Reardon et al. [22] for SSA. The latter is better suited for our data as compared to the NOVA classification system to describe dietary behavior characterized by different food processing levels in rural Kenya, Tanzania, and Uganda. Specifically, this study aims to: (a) ascertain the level of overweight and obesity among rural women in East Africa (Kenya, Tanzania, and Uganda); (b) approximate patterns in rural East Africa as explained by different food processing levels; and (c) analyze the relationship between the patterns characterized by the different food processing levels, with overweight and obesity as health outcomes. In all three countries, only women were recruited because women play an important role in shaping household nutrition—by way of caregiving—especially in rural areas, and especially for children. Hence, studying their dietary behavior is imperative to be able to design appropriate interventions that could shape the entire household nutrition [23].

This study is integrated in an overall project named “Fruits and Vegetables for all seasons” (FruVase). The project seeks to promote improved resource-efficient processing techniques and new market solutions for surplus fruits and vegetables for rural development in Kenya, Tanzania, and Uganda.

## 2. Materials and Methods

### 2.1. Study Areas and Design

Under the FruVase project, seven study sites were chosen to study six specific nutritious fruits and vegetables. Kenya consisted of the Taita-Taveta and Kitui counties to study guava fruit (*Psidium guajava*) and cowpea leaves (*Vigna unguiculata*), respectively. In Tanzania, cashew apple (*Anacardium occidentale)* was studied in Mtwara, while African nightshade (*Solanum* spp.) was researched on in Morogoro. The Kayunga, Jinja, and Kasese districts were selected to study jackfruit (*Artocarpus heterophyllus)*, cowpea leaves (*Vigna unguiculata*), and cassava leaves (*Manihot esculenta*), respectively. In these selected areas, various forms of malnutrition exist (Table 1) despite the excellent nutrient sources of the fruits and vegetables indicated above. All the study sites were selected for this sub-study within the project except for Kasese in Uganda, due to an Ebola outbreak during the data collection.

Two sampling designs were employed: purposive and simple random. Sub-regions, districts, or counties in the study areas that frequently cultivate the selected fruits and vegetables, which were, hence, available for consumption, were purposively chosen. In Tanzania, two sub-regions were selected per area, one sub-district per district was chosen in Uganda, and three sub-counties were selected in Kenya: two in Taita-Taveta and one in Kitui. A list of communities/villages for the sub-areas was obtained from the regional, district, and county offices. Per study area (six areas), 10 communities/villages were randomly selected. A comprehensive list of households in the randomly selected communities/villages was compiled with the help from community health workers/nutritionists and community leaders. Inclusion criteria were households with a woman of reproductive age (15–49 years) excluding pregnant women, and a child between 6 and 23 months, and/or a school-aged child between 6 and 13 years. Thirty households were randomly chosen per community/village except for those in Morogoro in Tanzania and Kayunga in Uganda. In these two areas, between 21 and 47 and 20 and 49 households were selected per community/village, respectively, proportional to the number of households (see Figure S1). During the survey, neighboring households that met the inclusion criteria were selected in instances where a sampled household did not meet the criteria, or the woman could not be reached. Overall, 1800 households were sampled across the three countries—600 households per country. Since the focus of the present study is on women, we only used and discussed the data applicable to the women.

### 2.2. Data Collection

A combination of cross-sectional and longitudinal household surveys was conducted with the women via face-to-face interviews. Socio-economic and demographic data were collected through cross-sectional surveys. These data consisted of age, education (years spent schooling and level), household headship, occupation, nutrition education, marital status, household size, and wealth status using seven household variables including the type of building materials for house floor, wall, and roof, the availability of potable water, toilet facility, ownership of agricultural land, and livestock.

Again, the cross-sectional data included anthropometric measurements as indicated in Table 1. Weights and heights were measured following the measurement guidelines on anthropometry [32]. Measurements were taken twice and recorded for each woman to ensure accuracy. Final weights and heights measurements were obtained by averaging the recorded values. Electronic Seca scales calibrated to a minimum weight of 0.1 kg were used to determine weights. Women stood on the scale wearing light clothing and were barefoot. For height measurement, women stood on height boards, except in Kenya where stadiometers were used, and were barefoot and without any headgear. Measurements were recorded to the nearest 0.1 cm. During analysis, implausible measurements were screened for and excluded.

Dietary assessment (24-h dietary recall) was conducted using the longitudinal approach (Table 1). Data were collected across the plenty and lean seasons of the FruVase targeted fruits and vegetables. During the survey, all food and drinks consumed during the previous 24 h of the day of visit were collected using the 24-h dietary recall tool. Per season, two non-consecutive dietary recalls were conducted. Local plates/bowls and cups, as well as different sizes of typical local fruits and snacks, served as measurement units. For example, women were asked to identify how many plates/bowls of “ugali” (in Kenya or Tanzania) or “posho” (in Uganda), a stiff porridge, they consumed, and how many plates/bowls of a side dish or other accompanying foods. Additionally, if they consumed any fruit or snack, they were asked to identify the size and the number. For drinks, local cups were used to assess the quantity of consumption.

Standard recipes were prepared per country to ascertain the actual portion size of food and drinks collected during the survey. First, the quantity of each ingredient was weighed before cooking. Meal/dishes were then fitted in the local plates/bowls and cups, used as measurement units during the survey, and weighed (as a portion size, which was done twice, and the average was calculated). Conversion rates were calculated by dividing the entire quantity of prepared meal/dish by the portion size that fit into the local plates/bowls or cups. The quantity of ingredients of a meal/dish was calculated by dividing the ingredients (weighed before cooking) by the respective conversion rate. The actual portion size of intake for a meal/dish for a woman was calculated by multiplying the portion size (fitted into the local plates/bowls or cups) by the unit of measurement collected during the survey. Additionally, ingredient quantities for the actual portion size were computed by multiplying the ingredients of the fitted meal/dish by the respective unit of measurement collected during the survey. For instance, if a woman consumed two plates of “ugali” or “posho”, we multiplied two by the fitted portion size of “ugali” or “posho”. We also determined the quantity of ingredients (example, maize flour and water) by multiplying the ingredients (maize flour and water) of the fitted portion size of “ugali” or “posho” by two. Likewise, if a woman took five medium-sized sweet bananas, we multiplied five by the portion size of a medium-sized banana, as measured. These calculations are demonstrated mathematically below:

PSS_k_ = (local portion size 1 + local portion size 2)/2 (1)

CR_k_ = WM_k_/PSS_k_ (2)

IPSS_k_ = WI_k_/CR_k_ (3)

APS_kj_ = PSS_k_ × MU_kj_ (4)

IAPS_kj_ = IPSS_k_ × MU_kj_ (5)

where portion size for a meal/dish (fitted into a local plate/bowl or cup) = PSS_k_; conversion rate for a meal/dish = CR_k_; total weight/quantity of meal/dish cooked (in one pot) = WM_k_; ingredients for fitted portion size of meal/dish = IPSS_k_; weighed ingredients for a meal/dish before cooking = WI_k_; actual portion size of a meal/dish for a particular woman = APS_kj_; measurement unit collected during survey for a meal/dish or food item for a particular woman = MU_kj_; ingredients for actual portion size of a meal/dish for a particular woman = IAPS_kj_.

The standard recipes prepared were complemented by the Kenya food recipes [33] and the Tanzania food composition tables [34]. For statistical analysis, the ingredients/food items and their respective quantities of intake were largely used.

### 2.3. Extration of Patterns and Food Processing Levels

Patterns were approximated by means of PCA. Although other methods exist, PCA has been largely used for many studies linking patterns to health outcomes (see the Section 1). Hence, its adoption for this study provided an avenue for the comparability of results. PCA is a statistical tool that reduces a large number of, in this case, foods consumed in grams per day into a smaller group of factors that shows the combinations of different food groups consumed and can be used to explain dietary behavior of study subjects or a population [10,35]. This tool was therefore applied to convert food items from Kenya (63), Tanzania (68), Uganda (81), and East Africa (103)—as pooled data of the three countries—into different factors to characterize the dietary behavior of rural women.

First, food intake quantities for all four dietary recalls (two per season) were screened for outliers, and implausible quantities were removed. The quantities for the four recalls were aggregated. Food items—excluding condiments and spices—were then classified into food groups based on intake distribution, nutrient similarities, and their levels of processing. Overall, three food processing levels were defined: unprocessed, minimally processed, and highly processed. Unprocessed foods were foods that required simple cleaning or washing and had not been subjected to any form of processing; minimally processed foods had undergone some form of modifications such as drying, fermentation, milling, freezing, pasteurization etc.; and highly processed foods (including ultra-processed and ultra-processed prepared Foods Away From Home (FAFH)) were defined as foods with potentially high amount of salt, sugar, oil, and/or multiple ingredients that are manufactured by small and medium enterprises (SMEs) and industrial firms to extend their shelf-lives and make them attractive and palatable [22]. Food items were classified according to these three definitions under agriculture interpretation—where foods existed in their raw forms before being converted for consumption, and nutrition interpretation—where foods were converted through some preparation methods for final consumption. The nutrition interpretation was adopted for the final analysis. Tables S1–S4 indicate how the food items identified were classified based on the food processing definitions, and under the agriculture and nutrition interpretations for Kenya, Tanzania, Uganda, and East Africa, respectively. The food and drinks were initially classified into 20 food groups, then were re-classified into 14, 12, and 11 food groups for analysis and comparison. For each food group under the four classifications, the amount of intake (in grams) was obtained by summing up the intake amount of all food items identified under that food group. Intake quantity (in grams) per food group was then standardized—mean = 0 and standard deviation = 1—to account for large variances that may exist across the food groups [18].

The standardized values were fed into the PCA tool as primary data to extract patterns. Varimax with Kaiser Normalization was used as a rotation method to extract patterns that are not correlated to each other, but rather correlations between food groups that describe patterns [16,17,18]. After comparison, the 11-food grouping was chosen for Tanzania, Uganda, and East Africa, while the 12-food grouping was selected for Kenya. Rice was constructed as an additional food group in the Kenya food grouping. These groupings provided for the most detailed and coherent interpretation of patterns as well as the highest total percentage of variance explained in the patterns.

Following other studies [9,17,18], patterns with eigenvalues >1 and explained by a high percentage of variance were retained. In all, four patterns were retained, except for Uganda, where five were retained. Patterns were interpreted and labelled with food groups with rotated factor loadings ≥ ±0.45. Factor loadings showcase the correlation between the food groups and the patterns; high positive loadings indicating positive correlations and high negative loadings representing an inverse relationship. Factor scores were computed for each pattern by aggregating the intake amount of the standardized food groups weighted by their respective factor loadings. Finally, each woman obtained a factor score for each pattern: a high positive score indicating a woman’s close association with such pattern and a negative score indicating otherwise [15,17].

### 2.4. Statistical Analysis

Descriptive statistics by way of means were calculated for age, education (years spent in school), household head (yes = 1, no = 0), nutrition education (yes = 1, no = 0), household size, and body mass index (BMI). Significant differences across the countries were tested for using the Kruskal–Wallis and Dunnett tests. Percentage distributions were calculated for the variables: marital status, occupation, education level, and wealth status. BMI was calculated as weight (kg) divided by height (m) squared and classified as the following: underweight = <18.5 kg/m^2^; normal = 18.5–24.9 kg/m^2^; overweight = 25.0–29.9 kg/m^2^; and obesity = ≥30.0 kg/m^2^, according to the WHO report on obesity [36]. The association between socio-economic and demographic variables and patterns was determined using the Spearman and point-biserial correlation methods. The Spearman correlation (ρ) was applied to two non-normally distributed continuous variables, while the point-biserial (r_pb_) correlation was applied to one dummy variable and one continuous variable [37].

Factor scores of each pattern were divided into three terciles. The first tercile represents women within the lowest segment of a pattern, while those in the third tercile represent the highest segment of a pattern. Logit regression models were constructed to ascertain the association between overweight and obesity and patterns. In these models, overweight and obesity (yes = 1, no = 0) was the dependent variable and the pattern (in terciles) was the main explanatory variable. Subsequently, these models were adjusted for possible confounders using the socio-economic and demographic variables. Since coefficients of logit models are difficult to interpret, odds ratios (OR) and the 90% confidence interval (CI) were calculated for logical interpretation. With OR, the odds of women within the highest tercile of a pattern being overweight and obese relative to those in the lowest tercile of the same pattern were calculated. A linear trend across the terciles was tested using the continuous values of the patterns to denote significance [17], as shown in terciles 3.

To understand the relationship between patterns and BMI, the ordinary least square (OLS) model was employed. BMI values were used as dependent variable, while the factor scores of the pattern served as the independent variable in the OLS models. However, the residuals of the models were not normally distributed—violating a key assumption of an OLS model. The socio-economic and demographic variables were included in the models to obtain normally distributed residuals. These variables also acted as covariates. Possible multi-collinearity was tested using variance inflation factor (VIF), and robust standard errors were used to account for heteroscedasticity. Location as a dummy variable was included in both the logit and OLS models for East Africa to account for unobserved differences, such as culture and other socio-economic factors, across the three countries. Statistical significance was pegged at 10%, 5%, 1%, and 0.1%. Analyses were conducted for adult women ≥19 years, [32], and separately for Kenya (N = 445); Tanzania (N = 292); Uganda (N = 415); and East Africa (N = 1152) as pooled data, using the R statistical tool 4.0.0.

## 3. Results

### 3.1. Socio-Economic and Demographic Characteristics

The average age of the women in Kenya, Tanzania, and Uganda was 33.8, 32.2, and 31.3 years, respectively. There were significant differences between the average age in Kenya and the other two countries. Between Tanzania and Uganda, there was no significant difference. The mean age in East Africa was estimated at 32.5 years. The highest level of education for more than half of the women was primary school across the board. In East Africa, this culminated to an average of 7.5 years in school for a rural woman; however, there were significant differences across countries (Table 2). Overall, more than 70% were married, which reinforced the fact that most of them are not household heads. Low nutrition education (for the previous 6 months to the day of the survey) was recorded across countries. A relatively large proportion of the women lived in households classified as having high wealth. Further details are illustrated in Table 2.

### 3.2. Body Mass Index as a Proxy for Health Outcomes

**Figure 1:**
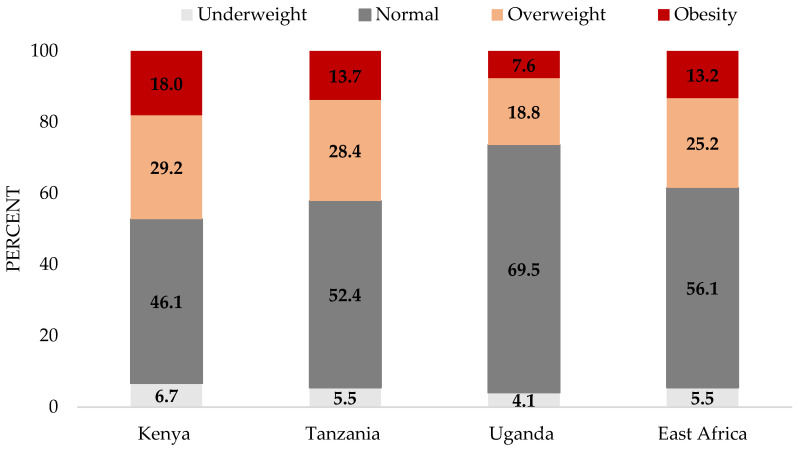
The rate of underweight, normal weight, overweight, and obesity among rural women of reproductive age in Kenya (N = 445), Tanzania (N = 292), Uganda (N = 415), and East Africa (N = 1152).

The BMI in East Africa among rural women was 24.5 kg/m^2^—almost at the threshold of overweight. In Kenya, the average BMI was within the overweight classification. Country comparison showed significant differences between Kenya and Uganda, and Tanzania and Uganda (Table 2). In Kenya and Tanzania, the rates of overweight and obesity were almost twice as much as Uganda. Overall, around 25% and 13% of the rural women were overweight and obese, respectively, in East Africa (Figure 1).

### 3.3. Patterns as Characterized by Food Processing Levels

Four components were retained to characterize diet behavior in Kenya and were labeled as plant-based, purchase I, plant- and animal-based, and purchase II patterns, respectively. The four patterns explained 59% of the variation in diets. The plant-based pattern was characterized by the intake of starchy plants such as cereals, roots, and tubers, as well as of pulses and nuts, and oils and fats—a mixture of minimally and highly processed foods. Components two and four were classified as purchase patterns because the foods under them were mostly purchased in supermarkets, small shops, and from street food vendors. They consisted of both minimally and highly processed foods (see Table 3). The plant- and animal-based pattern was made up of plant and animal food sources. Patterns identified in Kenya were also found in Tanzania. The difference, however, was that only one purchase pattern, described by highly processed foods, was identified in Tanzania. Additionally, a distinct pattern (starchy plants) made up of minimally processed cereals, roots, and tubers was found. The four patterns explained 60% of the variance in diets among rural women (Table 4).

In Uganda, three out of the five patterns—the purchase pattern, plant-based pattern I, and plant-based pattern II— retained can be related to the patterns found in Kenya and Tanzania. Just like in Tanzania, the purchase pattern in Uganda consisted wholly of highly processed foods. The two plant-based patterns reflected the plant-based and starchy plants patterns extracted in Kenya and Tanzania. The two additional patterns extracted were animal-based and vegetarian. The animal-based pattern was made up of poultry, fish, meat, and milk, while the vegetarian pattern consisted of vegetables (Table 5). Again, in Table 5, oils and fats correlated positively with these patterns, indicating that most of the animal food sources and vegetables were fried or at least consumed in combination with oils and fats in rural Uganda. For East Africa as a whole, four patterns, namely mixed, plant-based, purchase, and vegetarian, were identified. They cumulatively explained 53% of the variance, as shown in Table 6. Except for the mixed pattern, all the patterns were identical with the patterns from the three countries. However, some differences existed in the food sources. While the purchase pattern consisted of highly processed foods—bread and snacks and tea, the plant-based and vegetarian patterns were wholly made up of pulses and nuts and vegetables, respectively. The mixed pattern was a mixture of four food groups, and was characterized by unprocessed, minimally, and highly processed foods (Table 6).

### 3.4. Correlation between Patterns and Socio-Economic and Demographics Indicators

The plant-based pattern identified in Kenya showed a mild significant positive correlation with household size (ρ = 0.12) and being married (r_pb_ = 0.10), but was negatively correlated with the wealth index (ρ = −0.31), education (ρ = −0.25), and nutrition education (r_pb_ = −0.22). Purchase pattern II was positively correlated with the wealth index (ρ = 0.12) and education (ρ = 0.13), and, at the same time, was slightly negatively associated with household size (ρ = −0.15) and being married (r_pb_ = −0.09). Other correlation results for Kenya are detailed in Table S5. For Tanzania, the plant-based pattern was positively correlated with being married (r_pb_ = 0.11) and was negatively associated with the wealth index (ρ = −0.16). Just as it was observed in Kenya, the purchase pattern was positively correlated with the wealth index (ρ = 0.10) and education (ρ = 0.17), in addition to age (ρ = 0.12). Being a household head in Tanzania was positively associated with women following the plant- and animal-based pattern (r_pb_ = 0.13). In Table S6, starchy plants pattern correlated positively with age only (ρ = 0.19). 

For the correlation between the patterns obtained in Uganda and the socio-economic and demographic factors, the purchase pattern was positively correlated with the wealth index (ρ = 0.09) and education (ρ = 0.26), as observed in Kenya and Tanzania, while it was inversely associated with age (ρ = −0.15), being a household head (r_pb_ = −0.09), and household size (ρ = −0.10). Plant-based patterns I and II showed different associations: plant-based pattern I was positively correlated with education (ρ = 0.12) and nutrition education (r_pb_ = 0.19) and had a mild negative association with household size (ρ = −0.09); and plant-based pattern II was only positively correlated with age (ρ = 0.10) and was negatively correlated with the wealth index (ρ = −0.17). Further correlation results are shown in Table S7. In East Africa, the correlation results showed that purchase pattern (highly processed food-based diet) was positively correlated with the wealth index (ρ = 0.17) and education (ρ = 0.31) and had a weak relationship with nutrition education (r_pb_ = 0.07). This means as wealth, education, and nutrition education increase, more women follow the purchase pattern. Yet, there was a negative relationship with household size (ρ = −0.08). The plant-based pattern—characterized by pulses and nuts—correlated positively with age (ρ = 0.10), household size (ρ = 0.10), and education (ρ = 0.16). Thus, as age, household size, and education increase, women are more likely to follow the plant-based pattern (see Table S8).

### 3.5. Association between Patterns and Overweight and Obesity

**Figure 2:**
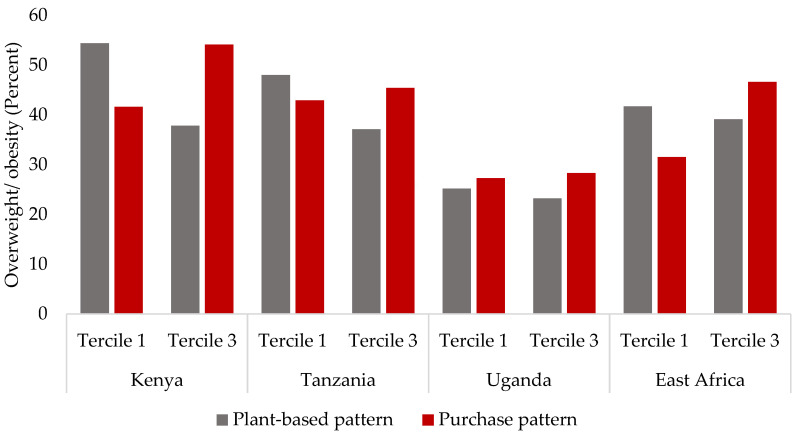
Percentage of overweight and obese rural women in the lowest (tercile 1) and highest (tercile 3) segments of the patterns (only plant-based and purchase patterns are shown here) in Kenya (N = 210), Tanzania (N = 123), Uganda (N = 110), and East Africa (N = 443). For Uganda, figures of plant-based pattern II are shown, while purchase pattern II is shown for Kenya.

Table 7 highlights the association between the patterns and overweight and obesity. In Kenya, there was a significant positive association between women in tercile 3 of purchase pattern II (bread and snacks and rice) and overweight and obesity (OR 1.52; CI 0.99, 2.32) relative to those in tercile 1. More than half (54%) of the overweight and obese women were within the highest segment (tercile 3) of purchase pattern II (refer to Figure 2). There was no significant association between the other patterns and overweight and obesity (Table 7). The same table shows that none of the patterns were significantly associated with overweight and obesity except for the plant-based pattern in Tanzania. Women within the highest tercile, following the plant-based pattern, compared to those at the lowest tercile of this pattern, were about 41% less probable of being overweight and obese (OR 0.59; CI 0.35, 0.98). Hence, the percentage of overweight and obese women within the highest tercile of this pattern was lower, as shown in Figure 2. None of the patterns were significantly associated with overweight and obesity in Uganda. For East Africa as a whole, only two patterns—the plant-based and purchase patterns—were significantly associated with overweight and obesity. The plant-based pattern was negatively associated with overweight and obesity (OR 0.59; CI 0.41, 0.85). On the other hand, the purchase pattern, characterized by highly processed foods—bread and snacks and tea—was positively associated with overweight and obesity. Women in the highest tercile were 22% more probable of being overweight and obese than women in the lowest tercile (OR 1.22; CI 0.89, 1.66). Overall, about almost half (46%) of the overweight and obese women were found in the highest tercile of the purchase pattern (Figure 2).

### 3.6. Relationship between Patterns and BMI (in Continous Values)

Table 8 showcases the relationship between the patterns and BMI across countries and East Africa. The two purchase patterns in Kenya were significant and positively associated with high BMI values. The plant-based pattern had an inverse relationship with BMI. Again, in Tanzania, the plant-based pattern was negatively associated with BMI, as shown in Table 8. No significant relationship was found between the patterns and BMI in Uganda. In East Africa, the purchase pattern, defined by highly processed foods, increased BMI, indicating a significant positive relationship between the two variables. In contrast, the plant-based pattern—characterized by pulses and nuts—reduced BMI, signaling a negative relationship between the two variables. Mixed and vegetarian patterns showed no significant relationship with BMI. Full models are shown in Tables S9–S12 for Kenya, Tanzania, Uganda, and East Africa, respectively.

## 4. Discussion

### 4.1. Overweight and Obesity in Sub-Saharan Africa

The present study demonstrated the current rates of overweight and obesity, patterns characterized by food processing levels, and how they are associated with overweight and obesity among rural women in East Africa. This study showed that the overweight and obesity rate is high in rural Kenya and Tanzania compared to Uganda. Regionally, the rate (overweight and obesity combined) was 38.4%. According to Shekar and Popkin [3] and Reardon et al. [22], overweight and obesity are rapidly rising in SSA particularly among individuals within the highest socio-economic strata, and hence, the high rates identified in the present study are not entirely surprising. Indeed, this is backed by other studies in SSA that have documented high rates of overweight and obesity. Ajayi et al. [38] in their study of four SSA countries, reported high rates of overweight and obesity in both urban and rural areas. For instance, the overweight and obesity rate was 46% in rural Uganda and 75% in urban Tanzania. In a study by Keding et al. [39], among women in rural Tanzania, 22% of the women were overweight and obese. Additionally, the Kenya Ministry of Health [40] in its mid-term review report of the health sector strategic plan in 2016 reported between 35% and 44% overweight and obesity rates for individuals residing in Taita-Taveta and Kitui counties (the same counties sampled for this study). The report also expressed concern of a continuous increase in overweight and obesity and the danger as a national health issue. The figure of overweight and obesity depicted in this study for Kenya (47%) was in line with that of the Kenya Ministry of Health [40]; for Tanzania (42%), it was almost twice the findings by Keding et al. [39]; yet, the data for the latter study were collected more than 10 years ago, which could explain the increase; and in rural Uganda, our result (26%) was almost half the rate indicated by Ajayi et al. [38], suggesting that the rates differ highly between different rural areas.

Over the years, overweight and obesity rates have risen among women globally. A global trend analysis between 1980 and 2013 showed an increased rate of 8% [41]. In SSA, for instance, being a woman is a significant predictor for being overweight and obese [38]. In a study by Holmes et al. [17], data pooled from four countries showed that overweight and obesity rates for women were more than twice as much as men. Similar statistics were reported in Burkina Faso [14] and Kenya [40]. Despite these findings being largely urban centered, Popkin et al. [5] intimated that rural women are also increasingly catching up with their compatriots in urban areas. The present study reaffirms this assertion made by Popkin et al. [5] of growing overweight and obesity among rural women. This unfolding trend is particularly worrying because women play a critical role in shaping the overall household nutrition especially in rural areas [23]. Moreover, bad health outcomes such as overweight and obesity for women are a huge public health concern that consequently poses adverse effects for the proper cognitive and healthy development of young children, and in general, for building a strong human capital base for a society.

### 4.2. Patterns and the Nutrition Transition

Patterns identified across the countries and at the regional level are not different and are not entirely different from patterns obtained in other studies by Keding et al. [15] and Auma et al. [18]. In rural Tanzania, Keding et al. [15] identified five patterns; the same number of patterns was extracted for Uganda and the four patterns extracted for Kenya, Tanzania, and East Africa equaled the number reported in the study of Auma et al. [18]. A purchase pattern, also identified by Keding et al. [15], was found in the three countries and at the regional level. Again, the purchase pattern reflected that of the transitioning, processed pattern reported in Uganda by Auma et al. [18]. Indeed, in these two studies and the present study, this pattern was made up of bread and snacks, sugar, tea (with milk, sugar, or both), rice, and pasta, which are sometimes considered micronutrient poor [42]. Identification of the purchase pattern across the study areas confirms the full force of the nutrition transition in rural areas. Additional patterns extracted, such as the plant-based, plant- and animal-based, and animal-based patterns, can be equated to the traditional inland and/or pulses, traditional coast, and animal product patterns in Keding et al. [15], respectively. To a large extent, the plant-based, traditional (high fat), and animal-based patterns described in Auma et al. [18] can also be reflected in the additional extracted patterns listed above. Aside from these similarities in patterns, a vegetarian pattern was uniquely identified in our study, but only in Uganda and East Africa. Although other patterns were identified, the trend across the countries shows a nutrition transition from stage 3 (“Industrialization/ Receding Famine”) to stage 4 (“Non-communicable Disease”) as defined by Popkin [43]. 

### 4.3. Processed Foods, Wealth, and Overweight and Obesity

The purchase pattern largely characterized by highly processed foods had a significant positive association with overweight and obesity and BMI in Kenya and East Africa, yet there was a non-significant negative association in Tanzania and Uganda. These mixed findings were also expressed by Togo et al. [10] in their review study when they identified heterogeneous (positive and negative) associations between patterns equivalent to the purchase pattern and obesity and BMI. Although the data are mixed, in the present study, there is a trend towards increased BMI values by following the purchase pattern. Generally, in SSA, there has been a rapid shift in dietary intake that prominently features the increased consumption of highly processed foods, which increases the risk of obesity and other nutrition-related diseases [22]. Unfortunately, their increased consumption also has adverse effects on the environment, social, and cultural dimensions: as the intensity of food processing increases, the energy required for processing and other activities such as packaging and transportation increases, and, additionally, traditional food cultures gradually diminish [44].

The increased consumption of highly processed foods has been attributed to varied factors including their easy accessibility even in small/local shops, high visibility through advertisement and campaigns, convenience, palatability, lower prices relative to fresh or minimally processed foods, and long shelf-life [19,42,45]. High income or wealth also has been identified as a trigger of increased intake of highly processed foods [42]. The authors of [42] further espoused that as disposable income increases, the consumption of highly processed foods increases, while the consumption of fresh or minimally processed foods decreases. Keding et al. [15] and the Kenya Ministry of Health [40] supported this notion in their studies, which determined that wealth has a direct relationship with the consumption of micronutrient-poor diets that are correlated with obesity and high BMI in Tanzania and Kenya, respectively. This trend is in accordance with the findings of our study, as the wealth index was significantly positively correlated with the purchase pattern—largely characterized by highly processed foods—and negatively correlated with patterns mainly characterized by unprocessed and minimally processed foods across the three countries and in East Africa. According to Reardon et al. [22], most rural folks who work constantly purchase food outside the home to reduce the opportunity cost of cooking at home. This could be applicable to our study areas, as most of the women were farmers and possibly spend more time working on the farm, and hence, constantly purchase food. In SSA, SMEs are responsible for 80% of minimally and highly processed and packaged foods that are manufactured domestically—such as mandazi and bread, also identified in this study. Hence, this can explain the apparent proliferation of highly processed foods on retail markets [22]. In Windhoek, Namibia, street food stands, small stores, mobile food vendors, and shops in open markets retail highly processed foods like snacks, oily foods, and sweetened beverages [46]. In rural Tanzania, small shops are active vehicles for selling highly processed foods like cookies [22]. It can be concluded that same street food vendors and small/local shops in our study areas could play an active role in the high consumption of highly processed foods, and consequently, the increased risk of overweight and obesity. Therefore, the so-called built food environment including both informal and formal markets [47] needs to be analyzed more carefully in relationship to health outcomes. 

The prevalence of obesity has raised negative publicity for processed foods, in general. Meanwhile, food processing can provide significant interventions for the continued flow of nutritious foods, and thus, not all processed and even highly processed foods are unhealthy [19,48]. Indeed, the findings of the present study suggest this also: the pattern characterized by largely minimally processed foods had a negative or no association with overweight and obesity and BMI, and the pattern mainly characterized by highly processed foods had a heterogenous association—positive or no association—with overweight and obesity and BMI. Both minimally and highly processed foods can provide the calorie and micronutrient needs for many poor households, who otherwise would suffer from hunger and micronutrient deficiencies due to high food perishability and food scarcity [48]. Previous studies have highlighted the importance of processed foods in diets: for example, frozen, dried, or canned fruits and vegetables contributed 35% of dietary fiber, 62% of vitamin E, 51% of vitamin C, 40% of folate, and 25% of vitamin A to diets in the USA [49]. Additionally, processed cereals fortified with iron, folate, and thiamine, and milk fortified with vitamin A and D, were used to reduce significantly the number of individuals with an inadequate intake of the above micronutrients [50]. Again, minimally processed fruits and vegetables, from the FruVase project, in the form of vegetable soup mix, dried vegetables, vegetable powder, and fruit and nut bars not only helped address micronutrients gaps of iron, zinc, and vitamin A and C in diets, but also substantially reduced diet cost by up to 53% [51]. In the present study, the underweight women (although small percentage) could rely on both minimally and highly processed foods to meet their nutrients needs. Additionally, women with high likelihood of being underweight during periods of food scarcity could depend on these foods to avert such a phenomenon. It must, however, be noted that highly processed foods enriched and fortified with nutrients could still have a low-calorie quality due to the loss of the food matrix during processing, and thus may not prevent the occurrence of chronic diseases [52]. 

### 4.4. Country Comparison

It was no surprise that none of the patterns was significantly associated with overweight and obesity and BMI in Uganda. First, Uganda recorded the lowest rate of overweight and obesity, almost half the rate as in Kenya or Tanzania, which might be a contributing factor. Additionally, food variety was highest in Uganda (81 food items), which could translate into high dietary diversity compared to the other countries. Rutayisire et al. [53] found no linkage between patterns and overweight and obesity among 8900 Chinese children, attributing overweight and obesity to possible triggers of genes, screening time, and unobserved environmental factors. These triggers could also account for the overweight and obesity recorded in Uganda, as well as in Tanzania for the non-significant association with the purchase pattern. Again, the non-significant association between the purchase pattern and overweight and obesity and BMI in Tanzania and Uganda relative to Kenya could also be attributed to differences in nutrients and the amount of salt, sugar, fat, and other additives in the foods, emphasizing that not all highly processed foods might be unhealthy, particularly in rural areas where industrially processed foods could be few. By analyzing the built food environments in the study areas, Kenya possessed more formal food markets than informal in the form of supermarkets, hypermarkets, and mobile vendors as compared to Tanzania and Uganda, where street food vendors, kiosks/small shops, and mobile food vendors were predominant relative to formal food markets. This could also be a significant contributing factor for the differences, as formal food markets like supermarkets have been shown to contribute to increasing overweight and obesity in adults [48].

### 4.5. Study Strengths and Limitations

One major strength of this study was the design and usage of a single methodological approach that was adapted to the local contexts of the three countries and provided a strong basis to compare the results. Additionally, the usage of longitudinal food intake data from all three countries is one strength that deserves mention; most studies thus far have used cross-sectional food intake data. We performed individual country and regional analyses with an adequate sample size. Additionally, we determined patterns with a clear distinction of food processing levels and how they affect overweight and obesity and BMI in rural areas– to our knowledge, this is the first study to do so for a rural setting in SSA.

A key limitation was the exclusion of physical activity as a confounder in our models, especially when previous studies [54,55] show that physical activity reduces the probability of obesity. Energy intake was also not adjusted for; however, energy adjustment does not reveal any significant results [56]. The usage of the 24-h recall to collect food intake data depends heavily on the knowledge and skills of the interviewer and is associated with recall bias [18]. Yet, this was mitigated by recruiting and training university students/graduates as enumerators from the food and nutrition fields as well as pre-testing and validating the 24-h recall tool together with other questionnaires. Furthermore, the four non-consecutive dietary recalls may have helped offset any intra-individual food intake variability. Although anthropometric measurements were cross-sectionally taken at different time periods within and across countries, no significant differences in BMI across seasons were envisaged, as only slight differences in BMI across seasons in rural areas exist [39]. This study was also limited by the sampling technique. Sampling was restricted to districts/counties/regions that produced selected fruits and vegetables that were focused on in the FruVase project, in which framework this study took place. Hence, the results may only be applicable to the selected sites, calling into question the external validity of the results. The PCA method has been criticized for its inability to generate patterns that act as risk factors to diseases [12]. Additionally, the subjective nature of its analytical procedure has been questioned, including food groupings, retained patterns, the factor loading coefficient, and pattern interpretation [18]. Notwithstanding these gaps, the PCA is a well-established method for dietary patterns research in SSA, and, as such, was useful for this study.

### 4.6. Policy Implications and Further Work

First, the seeming shift of dietary intake towards unhealthy processed foods in rural areas must be reversed. Reardon et al. [22] observed that increased risk of overweight and obesity was also because of no or low consumption of fruits and vegetables, legumes, and pulses. Therefore, a food environment where these nutritious foods are constantly advertised, available, and accessible must be pursued. The challenge with these highly nutritious foods is often seasonal availability (e.g., in terms of fruits and vegetables), where healthy processed options could bridge the gap. We propose investment in processing innovations and technologies, including research to preserve fresh and raw foods for a longer time, which would make foods available and at the same time affordable, especially for the poor. Furthermore, food-based dietary guidelines that would help shift consumption from unhealthy processed foods to healthy ones are needed for each country and should be adapted to the local situation. At present, in East Africa, only Kenya has food-based dietary guidelines [57].

Second, food manufacturers, street food vendors, and small shops retailing processed foods must be fully engaged at the national or sub-national levels when designing policies/interventions to curb unhealthy foods, and consequently, overweight and obesity levels. This is particularly important because portion size/calorie, as well as additive and ingredient control mechanisms can be pursued at that level to reduce the excessive consumption of unhealthy foods. While the definition of “unhealthy foods” must be clear on a local level, international benchmarks on the portion of intake of unhealthy processed foods should be enacted.

Taxes on highly processed foods help control obesity; however, caution is needed as these foods also provide the calorie needs of the poor [45]. We therefore propose that local taxes at the district/county/regional level can be increased for unhealthy processed foods, and the income be invested, used as subsidies, or as incentives for promoting and/or rewarding the production and processing of nutritious foods such as fruits and vegetables. Such a policy intervention could also spur employment through production and processing in rural areas.

One major challenge for processed foods is differentiating between nutritious/healthy and rather unhealthy choices, such as pure fruit juice *versus* fruit drinks with flavor and sugar or carefully solar-dried fruits or vegetables *versus* open sun-dried ones. The current food processing levels—of which there seems to be no consensus—that classify foods into healthy or unhealthy are problematic. Rather, the classification of processed foods regarding which are healthy or unhealthy must be based on the nutrients retained and inclusive of the quality of their calories, i.e., represented by a preserved food matrix effect, and the amount of added salts, sugar, fats, and other additives. As such, a comprehensive work on processed foods clearly defined as healthy or unhealthy based on some internationally or nationally agreed benchmarks on nutrients and the amount of salt, sugar, fat, and other additives must be done, which certainly requires an interdisciplinary approach of nutritionists, food scientists and technologists, food economists, and professionals from other food disciplines. In addition, an international consensus on food processing classifications with their standard criteria for inclusion of foods is needed—and most likely need to be reviewed every few years as new processing technologies and processed products evolve. Further work is needed to understand the attitude and behaviors of rural populations towards processed foods and overweight and obesity as well as their perception of different processed foods being healthy or unhealthy or both. Additionally, the consumption of heathy and unhealthy processed foods and their repercussions for the environment and economic activities in rural areas must be established in the literature. Panel data studies are needed to understand the trend of dietary intake and how that affects overweight and obesity across varied time periods.

## 5. Conclusions

This study showed rising overweight and obesity rates among rural women in East Africa. Several patterns characterized by three food processing levels were identified, yet two patterns—plant-based and purchase patterns—were dominant across Kenya, Tanzania, Uganda, and East Africa (as pooled data). The plant-based pattern, largely characterized by unprocessed and minimally processed foods, had an inverse or no association with overweight and obesity and BMI. On the other the hand, a purchase pattern mainly identified by highly processed foods had a positive or no association with overweight and obesity and BMI. These heterogenous findings indicate the need to draw clear distinctions on processed foods as healthy and unhealthy based on their nutrient provision, inclusive of the quality of calories, i.e., represented by a preserved food matrix effect, as well as the amount of added salt, sugar, fats, and other additives, to avoid their general mischaracterization as unhealthy. Nonetheless, policy interventions (see policy implications) must be instituted to shift the course of increased consumption of unhealthy processed foods towards nutritious fresh and/or processed foods such as fruits and vegetables, which consequently help curb overweight and obesity.

## Figures and Tables

**Table 1:** Survey timelines, data collected, and malnutrition indicators across the six rural study sites in Kenya, Tanzania, and Uganda.

Country	Study Area	FruVase Target Crop	Plenty Season		Lean Season		Malnutrition Indicators	References for Malnutrition Indicators
			Survey Date	Data Collected	Survey Date	Data Collected		
Kenya	Kitui	Cowpea leaves	December 2019	24-h recall	June 2019	24-h recall; anthropometrics; socio-economic and demographics	Stunting (35%) for young children	[24]
	Taita-Taveta	Guava	April/May 2019	24-h recall; anthropometrics; socio-economic and demographics	October 2019	24-h recall	48% food insecure;Stunting (35%) for young children.	[25,26]
Tanzania	Morogoro	African nightshade	June/July 2019	24-h recall; anthropometrics; socio-economic and demographics	January 2020	24-h recall	Iron deficiency for young children (66%) and women (47%).	[27,28]
	Mtwara	Cashew apple	October/November 2019	24-h recall	April/May 2019	24-h recall; anthropometrics; socio-economic and demographics	Iron deficiency for young children (59%)	[28,29]
Uganda	Jinja	Cowpea leaves	February 2020	24-h recall	August 2019	24-h recall; anthropometrics; socio-economic and demographics	Stunting (31%); wasted (2%); underweight (12%) for young children	[30]
	Kayunga	Jackfruit					Triple burden of malnutrition among women	[31]

Ten villages were chosen per study area. Total number of households: 300 per study area, 600 per country, and 1800 for the overall study.

**Table 2:** Socio-economic and demographic statistics of women of reproductive age in Kenya, Tanzania, Uganda, and East Africa.

Variables	Kenya	Tanzania	Uganda	East Africa (Pooled Data)
Age of participants (years)	33.88 *^KT^ (8.28)	32.24 (8.55)	31.25 ***^KU^ (8.46)	32.52 (8.46)
Household size	5.25 ***^KT^ (2.14)	4.62 ***^TU^ (1.94)	6.44 ***^KU^ (2.79)	5.52 (2.46)
Household head (yes = 1, no =0)	0.24 (0.43)	0.19 (0.39)	0.16 *^KU^ (0.37)	0.20 (0.40)
Body mass index (kg/m^2^)	25.35 (5.14)	24.87 ***^TU^ (4.80)	23.44 ***^KU^ (3.83)	24.54 (4.69)
Nutrition education received during the past 6 months (yes = 1, no = 0)	0.14 *^KT^ (0.35)	0.09 (0.28)	0.10 ^+KU^ (0.29)	0.11 (0.32)
Years spent in school	8.44 ***^KT^ (3.21)	6.54 **^TU^ (2.49)	7.21 ***^KU^ (3.65)	7.52 (3.31)
Educational level (%)				
None	3.82	8.90	8.89	6.94
Primary	67.42	79.79	54.33	65.83
Secondary	22.25	10.62	34.13	23.59
Tertiary	6.51	0.68	2.64	3.65
Marital status (%)				
Married	74.38	76.71	77.16	75.98
Widowed or divorced or single	25.62	23.29	22.84	24.02
Main occupation (%)				
None	15.06	7.88	17.79	14.22
Farmer	62.92	75.00	62.26	65.74
Trader	12.58	10.27	10.34	11.19
Other (vocational skills, civil servant, teacher)	9.44	6.85	9.61	8.84
Wealth status (%)				
Low	22.02	17.47	28.60	26.91
Medium	37.53	32.19	34.38	32.38
High	40.45	50.34	37.02	40.71
N	445	292	415	1152

Standard deviations are in parentheses. +, *, **,*** represent statistical significance of *p* < 0.1, *p* < 0.05, *p* < 0.01, *p* < 0.001 according to the Kruskal–Wallis and Dunnett tests for significant differences across countries. ^KT^: significant difference between Kenya and Tanzania; ^KU^: significant difference between Kenya and Uganda; ^TU^: significant difference between Tanzania and Uganda.

**Table 3:** Patterns extracted for women of reproductive age in Kenya based on 12 food groups and characterized by three food processing categories.

Rotated Component	Extracted Pattern	Food Group with Food Items	Loading Coefficient	Food Processing Level Explaining Pattern(in Nutrition)	Proportion Variance Explained (%)	Cumulative Variance Explained (%)
1	Plant-based pattern	Cereals, roots, and tubers: Irish potato, sweet potato, maize, sorghum, millet, taro, plantain	0.78	Minimally and highly processed	17	17
		Pulses and nuts: Beans, cowpea, green grams, peas	0.87			
		Milled cereals, roots, pulses, and nuts: Millet flour, maize flour, sorghum flour, wheat flour	−0.59			
		Oils and fats: Sunflower cooking oil, margarine, hydrogenated fat	0.48			
2	Purchase pattern I	Tea: tea	0.87	Minimally and highly processed	16	33
		Sugar and sugary drinks: Sugar, soda, sugary drinks (mango sweetened)	0.64			
		Milk: Pasteurized milk, fermented milk	0.68			
3	Plant and animal-based pattern	Vegetables: Amaranth leaves, African nightshade, cowpea leaves, cabbage, carrot, eggplant, kales, onion, spinach, tomato, bitter lettuce, pumpkin leaves	0.86	Minimally and highly processed	14	47
		Meat, poultry, and fish: Beef, poultry, goat meat, offals, small dried fish, fish, egg, pork	0.46			
		Oils and fats: Sunflower cooking oil, margarine, hydrogenated fat	0.72			
4	Purchase pattern II	Bread and snacks: Bread, chips, noodles, *mandazi*, *halfcake* (wheat dough fried in oil), *chapati* (wheat flatbread)	0.61	Minimally and highly processed	11	59
		**Rice:** rice	0.78			

Factor loadings ≥ ±0.45 are shown in the loading coefficient column to characterize the correlation between the food group and pattern.

**Table 4:** Patterns extracted for women of reproductive age in Tanzania based on 11 food groups and characterized by three food processing categories.

Rotated Component	Extracted Pattern	Food Group with Food Items	Loading Coefficient	Food Processing Level Explaining Pattern (in Nutrition)	Proportion Variance Explained (%)	Cumulative Variance Explained (%)
1	Plant and animal-based pattern	Vegetables: Amaranth leaves, African nightshade, cabbage, carrot, cassava leaves, Chinese cabbage, cowpea leaves, cucumber, eggplant, green pepper, jute mallow leaves, kales, okra, onion, potato leaves, pumpkin, pumpkin leaves, spinach, sweet potato leaves, tomato	0.85	Minimally and highly processed	19	19
		Meat, poultry, and fish: Beef, poultry, fish, goat meat, small dried fish	0.70			
		Oils and fats: Cashew nut milk, coconut milk, sunflower cooking oil	0.84			
2	Purchase pattern	Tea: tea	0.90	Highly processed	18	37
		Sugar and sugary drinks: Sugar, soda, sugary drinks (lemon, mango sweetened)	0.89			
		Bread and snacks: Bread, noodles, *mandazi*, *vitumbua* (dough from rice or maize or wheat flour fried in oil), buns, *chapati* (wheat flatbread), *samosa* (fried pastry with vegetables and/or meat filling)	0.52			
3	Plant-based pattern	Fruits: Cashew apple, jackfruit, lemon/orange, mango, passion fruit, pawpaw, watermelon, pineapple, sweet banana	0.63	Unprocessed and minimally processed	11	49
		Milled cereals, roots, pulses, and nuts: Cassava flour, groundnut flour/paste, maize flour, millet flour, rice flour	0.51			
		Pulses and nuts: Bambara groundnut, beans, cowpeas, groundnut	0.60			
4	Starchy plants	Cereals, roots, and tubers: Cassava, Irish potatoes, maize, plantain, rice, sweet potatoes, taro, millet	0.87	Minimally processed	11	60
		Bread and snacks: Bread, noodles, *mandazi*, *vitumbua* (dough from rice or maize or wheat flour fried in oil), buns, *chapati* (wheat flatbread), *samosa* (fried pastry with vegetables and/or meat filling)	−0.59			

Factor loadings ≥ ±0.45 are shown in the loading coefficient column to characterize the correlation between the food group and pattern.

**Table 5:** Patterns extracted for women of reproductive age in Uganda based on 11 food groups and characterized by three food processing categories.

Rotated Component	Extracted Pattern	Food Group with Food Items	Loading Coefficient	Food Processing Level Explaining Pattern (in Nutrition)	Proportion Variance Explained (%)	Cumulative Variance Explained (%)
1	Purchase pattern	Tea: coffee, tea	0.45	Highly processed	14	14
		Sugar and sugary drinks: Sugar, soda, sugary drinks (orange, passion, pineapple, tamarind sweetened)	0.74			
		Bread and snacks: Bread, buns, *mandazi*, *halfcake* (dough fried in oil), *chapati* (wheat flatbread), *bagiya* (cassava and soybean flour fried in oil), cake, chips, cornflakes, *hardcorn*, noodles, pancake, popcorn, *samosa* (fried pastry with vegetables and/or meat filling)	0.72			
2	Plant-based pattern I	Milled cereals, roots, pulses, and nuts: Beans flour, groundnut flour/paste, maize flour, millet flour, sesame paste, sorghum flour, soybean flour/paste	0.80	Minimally processed	13	27
		Pulses and nuts: Beans, groundnut, soybean	0.81			
3	Animal based pattern	Milk: Pasteurized milk	0.45	Minimally and highly processed	12	39
		Meat, poultry, and fish: Beef, poultry, smoked fish, egg, fish, goat meat, offals, small dried fish	0.80			
		Oils and fats: Sunflower cooking oil, ghee, palm oil	0.59			
4	Plant-based pattern II	Cereals, roots, and tubers: Taro, cassava, cassava flour, cocoyam, Irish potatoes, maize, millet, plantain, rice, sorghum, sweet potatoes	0.73	Unprocessed and minimally processed	11	51
		Fruits: Apple, avocado, guava, jackfruit, lemon/orange, mango, passion fruit, pawpaw, pineapple, sweet banana, watermelon	0.77			
5	Vegetarian pattern	Vegetables: African nightshade, amaranth leaves, bitter berries, bitter tomatoes, cowpea leaves, cucumber, cabbage, carrot, mushroom, eggplant, garden eggs, green pepper, kales, onion, pumpkin, pumpkin leaves, spinach, tomato	0.76	Minimally and highly processed	10	61
		Oils and fats: Sunflower cooking oil, ghee, palm oil	0.53			

Factor loadings ≥ ±0.45 are shown in the loading coefficient column to characterize the correlation between the food group and pattern.

**Table 6:** Patterns extracted for women of reproductive age in East Africa based on 11 food groups and characterized by three food processing categories.

Rotated Component	Extracted Pattern	Food Group with Food Items	Loading Coefficient	Food Processing Level Explaining Pattern (in Nutrition)	Proportion Variance Explained (%)	Cumulative Variance Explained (%)
1	Mixed pattern	Cereals, roots, and tubers: Cassava, cocoyam, maize, millet, plantain, Irish potatoes, rice, sorghum, sweet potatoes, taro	0.75	Unprocessed, minimally, and highly processed	17	17
		Fruits: Apple, avocado, cashew apple, guava, jackfruit, lemon/orange, mango, passion fruit, pawpaw, pineapple, sweet banana, tamarind, watermelon	0.48			
		Milk: Pasteurized milk, fermented milk	0.47			
		Sugar and sugary drinks: Sugar, soda, sugary drinks (lemon, mango, orange, passion, pineapple, tamarind sweetened)	0.76			
2	Plant-based pattern	Pulses and nuts: Bambara groundnut, beans, cowpeas, green grams, groundnut, peas, soybean	0.73	Minimally processed	13	30
		Milled cereals, roots, pulses, and nuts: Beans flour, cassava flour, groundnut flour/paste, maize flour, millet flour, rice flour, sesame paste, sorghum flour, soybean flour/paste, wheat flour	−0.49			
		Meat, poultry, and fish: Smoked fish, egg, fish, goat meat, offals, beef, poultry, pork, small-dried fish	−0.54			
3	Purchase pattern	Tea: tea, coffee	0.64	Highly processed	13	44
		Bread and snacks: Bread, buns, *mandazi*, *halfcake*, *vitumbua* (dough from rice or maize or wheat flour fried in oil), *chapati* (unleavened flatbread), *bagiya* (cassava and soybean flour fried in oil), chips, cornflakes, *hardcorn*, noodles, pancake, popcorn, *samosa* (fried pastry with vegetables and/or meat filling)	0.68			
4	Vegetarian pattern	Vegetables: African nightshade, amaranth leaves, bitter berries, bitter lettuce, bitter tomatoes, cabbage, carrot, cassava leaves, Chinese cabbage, cowpea leaves, cucumber, eggplant, garden eggs, green pepper, jute mallow leaves, kales, mushroom, onion, okra, sweet potato leaves, pumpkin, pumpkin leaves, spinach, tomato	0.81	Minimally processed	10	53

Factor loadings ≥ ±0.45 are shown in the loading coefficient column to characterize the correlation between the food group and pattern.

**Table 7:** Association between patterns extracted (in terciles) and overweight and obesity among rural women of reproductive age in Kenya, Tanzania, Uganda, and East Africa.

Patterns	Kenya		Tanzania		Uganda		East Africa	
	N	OR (90% CI)	N	OR (90% CI)	N	OR (90% CI)	N	OR (90% CI)
Plant-based pattern ^a^								
Tercile 1	149	1.00 (Ref.)	98	1.00 (Ref.)	139	1.00 (Ref.)	384	1.00 (Ref.)
Tercile 2	148	1.02 (0.66, 1.57)	97	0.79 (0.47, 1.31)	138	1.34 (0.84, 2.13)	384	0.71 (0.51, 0.99)
Tercile 3	148	0.72 (0.46, 1.12)	97	0.59 * (0.35, 0.98)	138	0.91 (0.56, 1.49)	384	0.59 ** (0.41, 0.85)
Purchase pattern ^b^								
Tercile 1	149	1.00 (Ref.)	98	1.00 (Ref.)	139	1.00 (Ref.)	384	1.00 (Ref.)
Tercile 2	148	1.13 (0.74, 1.72)	97	0.73 (0.44, 1.23)	138	0.78 (0.48, 1.26)	384	0.99 (0.74, 1.31)
Tercile 3	148	1.52 ^+^ (0.99, 2.32)	97	0.85 (0.51, 1.43)	138	0.95 (0.58, 1.53)	384	1.22 * (0.89, 1.66)
Plant and animal-based pattern								
Tercile 1	149	1.00 (Ref.)	98	1.00 (Ref.)	--	--	--	--
Tercile 2	148	0.96 (0.63, 1.44)	97	0.88 (0.54, 1.47)	--	--	--	--
Tercile 3	148	1.05 (0.68, 1.60)	97	0.87 (0.52, 1.47)	--	--	--	--
Purchase pattern I								
Tercile 1	149	1.00 (Ref.)	--	--	--	--	--	--
Tercile 2	148	0.88 (0.58, 1.34)	--	--	--	--	--	--
Tercile 3	148	0.98 (0.64, 1.49)	--	--	--	--	--	--
Starchy plants								
Tercile 1	--	--	98	1.00 (Ref.)	--	--	--	--
Tercile 2	--	--	97	1.23 (0.74, 2.05)	--	--	--	--
Tercile 3	--	--	97	0.76 (0.44, 1.28)	--	--	--	--
Vegetarian pattern								
Tercile 1	--	--	--	--	139	1.00 (Ref.)	384	1.00 (Ref.)
Tercile 2	--	--	--	--	138	0.96 (0.60, 1.54)	384	0.86 (0.66, 1.12)
Tercile 3	--	--	--	--	138	1.04 (0.64, 1.67)	384	0.76 (0.58, 1.00)
Plant-based pattern I								
Tercile 1	--	--	--	--	139	1.00 (Ref.)	--	--
Tercile 2	--	--	--	--	138	1.24 (0.77, 2.00)	--	--
Tercile 3	--	--	--	--	138	0.91 (0.56, 1.47)	--	--
Animal-based pattern								
Tercile 1	--	--	--	--	139	1.00 (Ref.)	--	--
Tercile 2	--	--	--	--	138	1.31 (0.82, 2.09)	--	--
Tercile 3	--	--	--	--	138	0.81 (0.50, 1.32)	--	--
Mixed pattern								
Tercile 1	--	--	--	--	--	--	384	1.00 (Ref.)
Tercile 2	--	--	--	--	--	--	384	0.69 (0.53, 0.90)
Tercile 3	--	--	--	--	--	--	384	0.96 (0.60, 1.57)

^a^ Plant-based pattern II for Uganda; ^b^ Purchase pattern II for Kenya; Tercile 1 = women within the lowest segment the pattern; Tercile 3 = women within the highest segment following the pattern. Models were estimated using logit regression modeling and odds ratio (OR) and confidence intervals at 90% were calculated. Overweight and obesity (binary) as outcome variable and pattern (in three terciles) as explanatory variable with socio-economic and demographic factors as covariates in the models. ^+^, *, **, represent the statistical significance of *p* < 0.1, *p* < 0.05, *p* < 0.01, respectively, for the trend test across the terciles.

**Table 8:** Relationship between extracted patterns and body mass index (BMI) of women of reproductive age in rural Kenya, Tanzania, Uganda, and East Africa.

Independent Variables	Body Mass Index (BMI)			
	Kenya	Tanzania	Uganda	East Africa
Plant-based pattern ^a^	−0.568 * (0.258)	−0.693 ** (0.257)	0.169 (0.166)	−0.696 *** (0.160)
Purchase pattern ^b^	0.422 ^+^ (0.256)	−0.243 (0.283)	−0.090 (0.180)	0.454 *** (0.137)
Plant- and animal-based pattern	−0.062 (0.282)	−0.131 (0.267)	--	--
Purchase pattern I	0.480 * (0.228)	--	--	--
Starchy plants	--	−0.200 (0.281)	--	--
Vegetarian pattern	--	--	−0.193 (0.118)	−0.117 (0.084)
Plant-based pattern I	--	--	−0.184 (0.157)	--
Animal-based pattern	--	--	−0.072 (0.153)	--
Mixed pattern	--	--	--	−0.180 (0.167)
Control Variables	Yes	Yes	Yes	Yes
N	445	292	415	1152

^a^ Plant-based pattern II for Uganda; ^b^ Purchase pattern II for Kenya. Models were estimated using ordinary least square regression modeling. Estimated coefficients and robust standard errors in parentheses are shown. Body mass index as an outcome variable and patterns as explanatory variables with socio-economic and demographic factors as control variables are shown. ^+^, *, **, ***, represent statistical significance of *p* < 0.1, *p* < 0.05, *p* < 0.01, *p* < 0.001, respectively.

## Data Availability

The data presented in this study are available on request from the corresponding author. The data are not publicly available due to data protection and ethical concerns.

## Supplementary Materials

The following are available online at https://www.mdpi.com/article/10.3390/nu13082866/s1, Figure S1: Sampling techniques used in selecting households for survey in Kenya, Tanzania, and Uganda; Table S1: Classification of food items identified in rural Kenya into food groups and processing levels; Table S2: Classification of food items identified in rural Tanzania into food groups and processing levels; Table S3: Classification of food items identified in rural Uganda into food groups and processing levels; Table S4: Classification of food items identified in rural East Africa into food groups and processing levels; Table S5: Correlation between dietary patterns extracted and socio-demographic indicators among rural women in Kenya; Table S6: Correlation between dietary patterns extracted and socio-demographic indicators among rural women in Tanzania; Table S7: Correlation between dietary patterns extracted and socio-demographic indicators among rural women in Uganda; Table S8: Correlation between dietary patterns extracted and socio-demographic indicators among rural women in East Africa; Table S9: Relationship between extracted patterns and body mass index (BMI) of women of reproductive age in rural Kenya; Table S10: Relationship between extracted patterns and body mass index (BMI) of women of reproductive age in rural Tanzania; **Table S11**: Relationship between extracted patterns and body mass index (BMI) of women of reproductive age in rural Uganda; Table S12: Relationship between extracted patterns and body mass index (BMI) of women of reproductive age in three countries in rural East Africa.
