# Supplementary material for: Dietary Patterns as Characterized by Food Processing Levels and Their Association with the Health Outcomes of Rural Women in East Africa
Source: Nutrients. 2021 Aug 20;13(8):2866. doi: 10.3390/nu13082866 (PMC8399242; doi:10.3390/nu13082866)
Supplement: Supplementary file 1 [file nutrients-13-02866-s001.zip › nutrients-1335340-supplementary.pdf]

Figure S1: Sampling techniques used in selecting households for survey in Kenya, Tanzania, and Uganda.

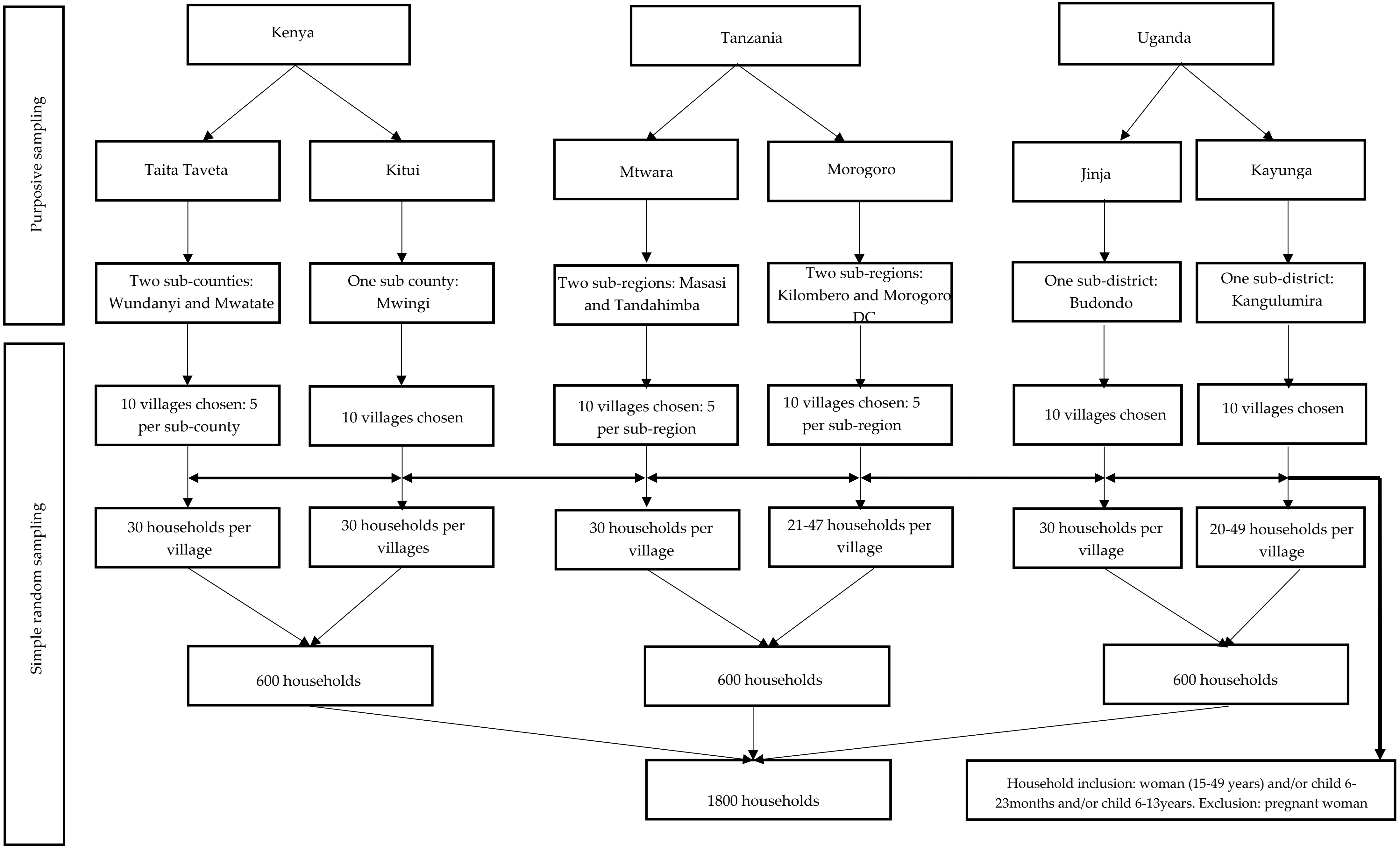

Table S1. Classification of food items identified in rural **Kenya** into food groups and processing levels.

Food group	Food items	Level of processing (in agriculture/form before consumption)	Level of processing (in nutrition/final form for consumption)[a]
Cereals, roots, and tubers	Irish potato, sweet potato, maize, sorghum, millet, taro, plantain	Unprocessed	Minimally processed
Vegetables	Amaranth leaves, African nightshade, cowpea leaves, cabbage, carrot, eggplant, kales, onion, spinach, tomato, bitter lettuce, pumpkin leaves	Unprocessed	Minimally processed
Fruits	Avocado, guava, mango, orange, passion fruit, pawpaw, pineapple, sweet banana, tamarind, watermelon	Unprocessed	Unprocessed
Pulses and nuts	Beans, cowpea, green grams, peas	Unprocessed	Minimally processed
Milled cereals, roots, pulses, and nuts	Millet flour, maize flour, sorghum flour, wheat flour	Minimally processed	Minimally processed
Rice	Rice	Minimally processed	Minimally processed
Meat, poultry, and fish	Beef, poultry, goat meat, offals, small-dried fish, fish, egg, pork	Minimally processed	Minimally processed
Milk	Pasteurized milk, fermented milk	Minimally processed	Minimally processed
Oils and fats	Sunflower cooking oil, margarine, hydrogenated fat	Highly processed	Highly processed
Sugar and sugary drinks	Sugar, soda , sugary drink(mango sweetened)	Highly processed	Highly processed

Bread and snacks	Bread, chips, noodles, *mandazi, halfcake* (dough fried in oil), *chapati* (wheat flatbread)	Highly processed	Highly processed
Tea	Tea	Highly processed	Highly processed

[a] Food items are converted for final consumption mainly through preparation methods such as boiling, frying, steaming, baking.

Table S2. Classification of food items identified in rural **Tanzania** into food groups and processing levels.

Food group	Food items	Level of processing (in agriculture/form before consumption)	Level of processing (in nutrition/final form for consumption)[a]
Cereals, roots, and tubers	Cassava, Irish potatoes, maize, plantain, rice, sweet potatoes, taro, millet	Unprocessed	Minimally processed
Vegetables	Amaranth leaves, African nightshade, cabbage, carrot, cassava leaves, Chinese cabbage, cowpea leaves, cucumber, eggplant, green pepper, jute mallow leaves, kales, okra, onion, potato leaves, pumpkin, pumpkin leaves, spinach, sweet potato leaves, tomato	Unprocessed	Minimally processed
Fruits	Cashew apple, jackfruit, lemon/orange, mango, passion fruit, pawpaw, watermelon, pineapple, sweet banana	Unprocessed	Unprocessed
Pulses and nuts	Bambara groundnut, beans, cowpeas, groundnut	Unprocessed	Minimally processed
Milled cereals, roots, pulses, and nuts	Cassava flour, groundnut flour/paste, maize flour, millet flour, rice flour	Minimally processed	Minimally processed
Milk	Pasteurized milk	Minimally processed	Minimally processed
Meat, poultry, and fish	Beef, poultry, fish, goat meat, small-dried fish	Minimally processed	Minimally processed

Oils and fats	Cashew nut milk, coconut milk, sunflower cooking oil	Highly processed	Highly processed
Sugar and sugary drinks	Sugar, soda , sugary drinks(lemon, mango sweetened)	Highly processed	Highly processed
Bread and snacks	Bread, noodles, *mandazi, vitumbua* (dough from rice or maize or wheat flour fried in oil), buns, *chapati* (wheat flatbread), *samosa* (fried pastry with vegetables and/or meat filling)	Highly processed	Highly processed
Tea	Tea	Highly processed	Highly processed

[a] Food items are converted for final consumption mainly through preparation methods such as boiling, frying, steaming, baking.

Table S3. Classification of food items identified in rural **Uganda** into food groups and processing levels.

Food group	Food items	Level of processing (in agriculture/form before consumption)	Level of processing (in nutrition/final form for consumption)[a]
Cereals, roots, and tubers	Taro, cassava, cassava flour, cocoyam, Irish potatoes, maize, millet, plantain, rice, sorghum, sweet potatoes	Unprocessed	Minimally processed
Vegetables	African nightshade, amaranth leaves, bitter berries, bitter tomatoes, cowpea leaves, cucumber, cabbage, carrot, mushroom, eggplant, garden eggs, green pepper, kales, onion, pumpkin, pumpkin leaves, spinach, tomato	Unprocessed	Minimally processed
Fruits	Apple, avocado, guava, jackfruit, lemon/orange, mango, passion fruit, pawpaw, pineapple, sweet banana, watermelon	Unprocessed	Unprocessed
Pulses and nuts	Beans, groundnut, soybean	Unprocessed	Minimally processed
Milled cereals, roots, pulses, and nuts	Beans flour, groundnut flour/paste, maize flour, millet flour, sesame paste, sorghum flour, soybean flour/paste	Minimally processed	Minimally processed

Milk	Pasteurized milk	Minimally processed	Minimally processed
Meat, poultry, and fish	Beef, poultry, smoked fish, egg, fish, goat meat, offals, small-dried fish	Minimally processed	Minimally processed
Oils and fats	Sunflower cooking oil, ghee, palm oil	Highly processed	Highly processed
Sugar and sugary drinks	Sugar, soda , sugary drinks (orange, passion, pineapple, tamarind sweetened)	Highly processed	Highly processed
Bread and snacks	Bread, buns, *mandazi, halfcake* (dough fried in oil), *chapati* (wheat flatbread), *bagiya* (cassava and soybean flour fried in oil), cake, chips, cornflakes, *hardcorn*, noodles, pancake, popcorn, *samosa* (fried pastry with vegetables and/or meat filling)	Highly processed	Highly processed
Tea	Tea, coffee	Highly processed	Highly processed

[a] Food items are converted for final consumption mainly through preparation methods such as boiling, frying, steaming, baking.

Table S4. Classification of food items identified in rural **East Africa** into food groups and processing levels.

Food group	Food items	Level of processing (in agriculture/form before consumption)	Level of processing (in nutrition/final form for consumption)[a]
Cereals, roots, and tubers	Cassava, cocoyam, maize, millet, plantain, Irish potatoes, rice, sorghum, sweet potatoes, taro	Unprocessed	Minimally processed
Vegetables	African nightshade, amaranth leaves, bitter berries, bitter lettuce, bitter tomatoes, cabbage, carrot, cassava leaves, Chinese cabbage, cowpea leaves, cucumber, eggplant, garden eggs, green pepper, jute mallow leaves, kales, mushroom, onion, okra, sweet potato leaves, pumpkin, pumpkin leaves, spinach, tomato	Unprocessed	Minimally processed

Fruits	Apple, avocado, cashew apple, guava, jackfruit, lemon/orange, mango, passion fruit, pawpaw, pineapple, sweet banana, tamarind, watermelon	Unprocessed	Unprocessed
Pulses and nuts	Bambara groundnut, beans, cowpeas, green grams, groundnut, peas, soybean	Unprocessed	Minimally processed
Milled cereals, roots, pulses, and nuts	Beans flour, cassava flour, groundnut flour/paste, maize flour, millet flour, rice flour, sesame paste, sorghum flour, soybean flour/paste, wheat flour	Minimally processed	Minimally processed
Milk	Pasteurized milk, fermented milk	Minimally processed	Minimally processed
Meat, poultry, and fish	Smoked fish, egg, fish, goat meat, offals, beef, poultry, pork, small-dried fish	Minimally processed	Minimally processed
Oils and fats	Cashew nut milk, coconut milk, sunflower cooking oil, hydrogenated fat, ghee, margarine, palm oil	Highly processed	Highly processed
Sugar and sugary drinks	Sugar, soda , sugary drinks (lemon, mango, orange, passion, pineapple, tamarind sweetened)	Highly processed	Highly processed
Bread and snacks	Bread, buns, *mandazi, halfcake, vitumbua* (dough from rice or maize or wheat flour fried in oil), *chapati* (wheat flatbread), *bagiya* (cassava and soybean flour fried in oil), chips, cornflakes, hardcorn, noodles, pancake, popcorn, *samosa* (fried pastry with vegetables and/or meat filling), vitumbua	Highly processed	Highly processed
Tea	Tea, coffee	Highly processed	Highly processed

[a] Food items are converted for final consumption mainly through preparation methods such as boiling, frying, steaming, baking.

Table S5. Correlation between dietary patterns extracted and socio-demographic indicators among rural women in **Kenya.**

Socio-demographic variables	Plant-based pattern	Purchase pattern I	Plant and animal-based pattern	Purchase pattern II
Age	-0.00	0.01	0.02	-0.00
Wealth index	-0.31***	0.04	0.06	0.12**
Household head (1=yes, 0=no)	-0.07	-0.14**	-0.08+	0.02
Household size	0.12*	0.06	-0.09+	-0.15**
Education (in years)	-0.25***	0.07	0.11*	0.13**
Marital status (1=married, 0= single or divorced or widowed)	0.10*	0.06	-0.08+	-0.09+
Nutrition education (1=yes, 0=no)	-0.22***	-0.04	0.16***	0.02
N	445	445	445	445

Spearman correlation method was used for age, wealth index, household size and years in school and the dietary patterns. Point-biserial method was used for household head, marital status, and nutrition education and the dietary patterns. +,*,**,***, represent statistical significance of $p < 0.1$, $p < 0.05$, $p < 0.01$, $p < 0.001$, respectively.

Table S6. Correlation between dietary patterns extracted and socio-demographic indicators among rural women in **Tanzania.**

Socio-demographic variables	Plant and animal-based pattern	Purchase pattern	Plant-based pattern	Starchy plants
Age	-0.04	0.12*	0.08	0.19**
Wealth index	0.09	0.10+	-0.16**	-0.05
Household head (1=yes, 0=no)	-0.13*	0.08	-0.00	0.09
Household size	0.03	-0.08	-0.03	0.07
Education (in years)	-0.06	0.17**	-0.04	0.09
Marital status (1=married, 0= single or divorced or widowed)	-0.08	0.01	0.11+	-0.01
Nutrition education (1=yes, 0=no)	0.06	0.05	0.02	0.07
N	292	292	292	292

Spearman correlation method was used for age, wealth index, household size and years in school and the dietary patterns. Point-biserial method was used for household head, marital status, and nutrition education and the dietary patterns. +,*,**, represent statistical significance of $p < 0.1$, $p < 0.05$, $p < 0.01$, respectively.

Table S7. Correlation between dietary patterns extracted and socio-demographic indicators among rural women in **Uganda.**

Socio-demographic variables	Purchase pattern	Plant-based pattern I	Animal based pattern	Plant-based pattern II	Vegetarian pattern
Age	-0.15**	-0.06	-0.09+	0.10*	-0.09+
Wealth index	0.09+	-0.00	0.03	-0.17***	-0.01
Household head (1=yes, 0=no)	-0.09+	0.06	-0.02	0.01	-0.07

Household size	-0.10*	-0.09+	0.11*	0.08	0.09+
Education (in years)	0.26***	0.12*	0.13**	-0.06	0.02
Marital status (1=married, 0= single or divorced or widowed)	-0.01	-0.00	-0.02	0.02	0.00
Nutrition education (1=yes, 0=no)	0.03	0.19***	0.12*	-0.03	0.13**
N	415	415	415	415	415

Spearman correlation method was used for age, wealth index, household size and years in school and the dietary patterns. Point-biserial method was used for household head, marital status, and nutrition education and the dietary patterns. +,*,**,***, represent statistical significance of $p < 0.1$, $p < 0.05$, $p < 0.01$, $p < 0.001$, respectively.

Table S8. Correlation between dietary patterns extracted and socio-demographic indicators among rural women in **East Africa.**

Socio-demographic variables	Mixed pattern	Plant-based pattern	Purchase pattern	Vegetarian pattern
Age	-0.07*	0.10***	0.01	0.02
Wealth index	-0.09**	-0.01	0.17***	-0.00
Household head (1=yes, 0=no)	-0.07*	0.03	-0.04	-0.05+
Household size	0.24***	0.10***	-0.08**	0.00
Education (in years)	-0.00	0.16***	0.31***	0.04
Marital status (1=married, 0= single or divorced or widowed)	0.05+	0.03	-0.04	-0.03
Nutrition education (1=yes, 0=no)	-0.01	-0.00	0.07*	0.05
N	1152	1152	1152	1152

Spearman correlation method was used for age, wealth index, household size and years in school and the dietary patterns. Point-biserial method was used for household head, marital status, and nutrition education and the dietary patterns. +,*,**,***, represent statistical significance of $p < 0.1$, $p < 0.05$, $p < 0.01$, $p < 0.001$, respectively.

Table S9. Relationship between extracted patterns and body mass index (BMI) of women of reproductive age in rural **Kenya**.

	Body mass index (BMI)			
Independent variables	(1)	(2)	(3)	(4)
Plant-based pattern	-0.568* (0.258)	--	--	--
Purchased pattern I	--	0.480* (0.228)	--	--
Plant and animal-based pattern	--	--	-0.062 (0.282)	--
Purchased pattern II	--	--	--	0.422+ (0.256)
Age	0.170*** (0.031)	0.177*** (0.032)	0.179*** (0.032)	0.173*** (0.032)
Wealth index	2.197***	2.415***	2.491***	2.436***

	(0.582)	(0.554)	(0.559)	(0.554)
Household head (1=yes, 0=no)	-1.095	-0.862	-1.059	-1.005
	(0.694)	(0.693)	(0.702)	(0.695)
Household size	0.011	-0.006	-0.002	0.025
	(0.115)	(0.115)	(0.116)	(0.116)
Education (in years)	0.007	0.024	0.020	0.013
	(0.079)	(0.077)	(0.079)	(0.077)
Marital status (1=married, 0= single or divorced or widowed)	0.134	0.120	0.065	0.144
	(0.677)	(0.681)	(0.696)	(0.691)
Occupation:				
None	0.761	1.168	1.071	0.767
	(0.813)	(0.815)	(0.807)	(0.786)
Trader	1.287^{+}	1.264	1.287	1.268
	(0.778)	(0.771)	(0.784)	(0.777)
Other	0.092	-0.171	0.109	0.088
	(0.763)	(0.754)	(0.784)	(0.774)
Nutrition education (1=yes, 0=no)	0.977	1.236	1.238	1.272^{+}
	(0.776)	(0.750)	(0.779)	(0.767)
Model intercept	19.206^{***}	18.808^{***}	18.824^{***}	18.940^{***}
	(1.440)	(1.444)	(1.446)	(1.435)
F-statistic	8.854^{***}	8.388^{***}	8.100^{***}	8.224^{***}
Adjusted R^2	0.15	0.14	0.14	0.14
N	445	445	445	445

Models were estimated using ordinary least square regression modeling. Estimated coefficients and robust standard errors in parentheses are shown. Body mass index as outcome variable and dietary pattern as explanatory variable with socio-economic factors as possible confounding factors. +, *, ***, represent statistical significance of p < 0.1, p < 0.05, p < 0.001, respectively.

Table S10. Relationship between extracted patterns and body mass index (BMI) of women of reproductive age in rural **Tanzania**

	Body mass index (BMI)			
Independent variables	(1)	(2)	(3)	(4)
Plant and animal-based pattern	-0.131	--	--	--
	(0.267)			
Purchase pattern	--	-0.243	--	--
		(0.283)		
Plant-based pattern	--	--	-0.693^{**}	--
			(0.257)	
Starchy pattern	--	--	--	-0.200
				(0.281)
Age	0.145^{***}	0.149^{***}	0.152^{***}	0.149^{***}
	(0.034)	(0.035)	(0.034)	(0.035)
Wealth index	0.817	0.851	0.518	0.751
	(0.671)	(0.675)	(0.668)	(0.678)
Household head (1=yes, 0=no)	-0.776	-0.686	-0.686	-0.680
	(0.828)	(0.811)	(0.798)	(0.820)
Household size	-0.097	-0.110	-0.121	-0.091

	(0.140)	(0.143)	(0.141)	(0.142)
Education (in years)	0.021	0.040	0.023	0.031
	(0.154)	(0.156)	(0.156)	(0.156)
Marital status (1=married, 0= single or divorced or widowed)	0.301	0.363	0.512	0.366
	(0.801)	(0.789)	(0.792)	(0.793)
Occupation:				
None	-1.493	-1.487	-1.444	-1.460
	(1.043)	(1.043)	(1.049)	(1.058)
Trader	1.108	1.147	1.020	1.220
	(0.826)	(0.819)	(0.812)	(0.827)
Other	-0.608	-0.677	-0.399	-0.596
	(1.425)	(1.397)	(1.430)	(1.425)
Nutrition education (1=yes, 0=no)	-0.087	-0.083	-0.047	-0.087
	(1.057)	(1.046)	(1.043)	(1.042)
Model intercept	20.492***	20.260***	20.194***	20.206***
	(1.523)	(1.543)	(1.526)	(1.615)
F-statistic	4.832***	4.878***	5.533***	4.916***
Adjusted R^2	0.08	0.08	0.10	0.08
N	292	292	292	292

Models were estimated using ordinary least square regression modeling. Estimated coefficients and robust standard errors in parentheses are shown. Body mass index as outcome variable and dietary pattern as explanatory variable with socio-economic factors as possible confounding factors. **,*** represent statistical significance of $p < 0.01$, $p < 0.001$, respectively.

Table S11. Relationship between extracted patterns and body mass index (BMI) of women of reproductive age in rural **Uganda**.

	Body mass index (BMI)				
Independent variables	(1)	(2)	(3)	(4)	(5)
Purchase pattern	-0.090	--	--	--	--
	(0.180)				
Plant-based pattern I	--	-0.184	--	--	--
		(0.157)			
Animal-based pattern	--	--	-0.072	--	--
			(0.153)		
Plant-based pattern II	--	--	--	0.169	--
				(0.166)	
Vegetarian pattern	--	--	--	--	-0.193
					(0.118)
Age	0.083***	0.084***	0.083***	0.082***	0.083***
	(0.024)	(0.024)	(0.024)	(0.024)	(0.024)
Wealth index	1.367**	1.349**	1.352**	1.400**	1.369**
	(0.446)	(0.441)	(0.441)	(0.443)	(0.441)
Household head (1=yes, 0=no)	-0.313	-0.249	-0.296	-0.289	-0.314
	(0.616)	(0.622)	(0.619)	(0.618)	(0.618)
Household size	0.107	0.103	0.111	0.104	0.109
	(0.077)	(0.078)	(0.079)	(0.079)	(0.078)

Education (in years)	0.045	0.047	0.043	0.039	0.043
	(0.060)	(0.059)	(0.058)	(0.058)	(0.058)
Marital status (1=married, 0= single or divorced or widowed)	1.155*	1.171*	1.150*	1.152*	1.157*
	(0.462)	(0.467)	(0.462)	(0.464)	(0.462)
Occupation:					
None	0.543	0.539	0.506	0.537	0.593
	(0.484)	(0.483)	(0.489)	(0.485)	(0.483)
Trader	1.250+	1.204+	1.186+	1.182+	1.201+
	(0.637)	(0.616)	(0.618)	(0.617)	(0.619)
Other	-0.011	0.013	-0.034	0.028	-0.041
	(-0.012)	(0.638)	(0.635)	(0.639)	(0.632)
Nutrition education (1=yes, 0=no)	-0.209	-0.102	-0.178	-0.195	-0.133
	(0.670)	(0.686)	(0.679)	(0.674)	(0.674)
Model intercept	18.816***	18.783***	18.835***	18.908***	18.814***
	(1.053)	(1.059)	(1.048)	(1.060)	(1.048)
F-statistic	3.182***	3.186***	3.184***	3.369***	3.426***
Adjusted R^2	0.07	0.07	0.07	0.07	0.08
N	415	415	415	415	415

Models were estimated using ordinary least square regression modeling. Estimated coefficients and robust standard errors in parentheses are shown. Body mass index as outcome variable and dietary pattern as explanatory variable with socio-economic factors as possible confounding factors. +,*,**,***, represent statistical significance of $p < 0.1$, $p < 0.05$, $p < 0.01$, $p < 0.001$, respectively.

Table S12. Relationship between extracted patterns and body mass index (BMI) of women of reproductive age in three countries rural **East Africa**

	Body mass index (BMI)			
Independent variables	(1)	(2)	(3)	(4)
Mixed pattern	-0.180	--	--	--
	(0.167)			
Plant-based pattern	--	-0.696***	--	--
		(0.160)		
Purchase pattern	--	--	0.454***	--
			(0.137)	
Vegetarian pattern	--	--	--	-0.117
				(0.084)
Age	0.138***	0.138***	0.138***	0.138***
	(0.017)	(0.017)	(0.017)	(0.017)
Wealth index	1.314***	1.279***	1.240***	1.333***
	(0.294)	(0.292)	(0.292)	(0.294)
Household head (1=yes, 0=no)	-0.622	-0.617	-0.539	-0.649
	(0.409)	(0.408)	(0.413)	(0.409)
Household size	0.024	0.023	0.026	0.020
	(0.058)	(0.058)	(0.057)	(0.058)
Education (in years)	0.056	0.048	0.041	0.052
	(0.046)	(0.045)	(0.045)	(0.045)
Marital status (1=married, 0= single or divorced or widowed)	0.663+	0.688+	0.682+	0.627+

	(0.367)	(0.367)	(0.369)	(0.366)
Occupation:				
None	0.634	0.516	0.555	0.658
	(0.424)	(0.416)	(0.418)	(0.424)
Trader	1.411***	1.358**	1.299**	1.371**
	(0.427)	(0.420)	(0.422)	(0.425)
Other	0.007	-0.062	-0.102	-0.020
	(0.498)	(0.495)	(0.494)	(0.498)
Nutrition education (1=yes, 0=no)	0.606	0.515	0.587	0.617
	(0.475)	(0.469)	(0.468)	(0.475)
Location:				
Tanzania	-0.133	-1.294**	0.254	-0.117
	(0.369)	(0.472)	(0.374)	(0.370)
Uganda	-1.174**	-1.936***	-1.030***	-1.457***
	(0.416)	(0.329)	(0.310)	(0.300)
Model intercept	19.279***	19.948***	19.256***	19.469***
	(0.839)	(0.799)	(0.798)	(0.797)
F-statistic	13.97***	15.02***	14.67***	14.21***
Adjusted R^2	0.13	0.14	0.13	0.13
N	1152	1152	1152	1152

Models were estimated using ordinary least square regression modeling. Estimated coefficients and robust standard errors in parentheses are shown. Body mass index as outcome variable and dietary pattern as explanatory variable with socio-economic factors as possible confounding factors. +,**,***, represent statistical significance of $p < 0.1$, $p < 0.01$, $p < 0.001$, respectively
